# Access to the 340B Drug Pricing Program: is there evidence of strategic hospital behavior?

**DOI:** 10.1186/s13104-021-05642-4

**Published:** 2021-06-03

**Authors:** Karen Mulligan, John A. Romley, Rebecca Myerson

**Affiliations:** 1 Sol Price School of Public Policy and Leonard D. Schaeffer Center for Health Policy & Economics, University of Southern California, 635 Downey Way, VPD Hall, Los Angeles, CA 90089 USA; 2 School of Pharmacy, University of Southern California, Los Angeles, USA; 3 Department of Population Health Sciences, University of Wisconsin-Madison, Madison, USA

**Keywords:** 340B, Hospitals, Strategic behavior, Policy

## Abstract

**Objective:**

The 340B Drug Pricing Program allows hospitals to purchase covered drugs at a discount and potentially generate profit if they are reimbursed at rates that exceed 340B acquisition prices. Disproportionate share hospitals (DSH) are eligible to participate in 340B if their DSH adjustment–a measure that identifies hospitals that treat a disproportionate share of low income Medicare or Medicaid patients–is above 11.75%. To assess whether hospitals behave strategically to gain access to the program, we examined data on the number of hospitals just above versus below the DSH adjustment threshold for 340B eligibility and conducted McCrary density tests to assess statistical significance.

**Results:**

In 2014–2016, the number of hospitals increases by 41% just above the 340B eligibility threshold. McCrary density tests found this increase to be statistically significant across a range of bandwidths in 2014–2016 (p < 0.01). From 2011–2013, the findings are sensitive to the bandwidth around the threshold, but insignificant in 2008–2010. We found no comparable change among hospitals ineligible for the 340B program. These data are consistent with the hypothesis that some hospitals adjust their DSH to gain 340B eligibility. Our findings support recent calls from the Government Accountability Office to improve oversight of the 340B program.

## Introduction

The 340B Drug Pricing Program was established in 1992 to enable covered entities, including hospitals, to stretch scarce federal resources by allowing them to purchase covered drugs at an estimated 20–50% discount [1]. Hospitals owned by state or local government or nonprofit hospitals with a contract with state or local government to provide healthcare services to low-income individuals who are not entitled to benefits under Medicare or Medicaid are eligible to participate in 340B [2, 3]. In addition to meeting this requirement, disproportionate share (DSH) hospitals must treat a “disproportionate” number of Medicaid patients. Specifically, to qualify for 340B, these hospitals must have a DSH adjustment percentage above 11.75% [4].

As noted by the US Government Accountability Office (GAO), hospitals can generate profit from 340B program participation by purchasing covered drugs at the discounted price for all patients and receiving reimbursement from insured patients that may exceed the 340B prices paid [5, 6]. Estimated per hospital profits from participating in 340B were $2.5 million in 2016, which accounts for a relatively small share of overall operating budgets [7]. Prior data suggest that US health care stakeholders adapt their behavior to incentives generated by the policy environment [8–11]. This study assessed the plausibility of strategic behavior to gain access to the 340B program by comparing the distribution of hospital DSH near the eligibility cutoff to what would be expected by chance alone, in the absence of strategic behavior.

## Main Text

### Methods

Our sample included US non-profit and public acute care hospitals with non-missing DSH information in the Healthcare Cost Report Information System (HCRIS) for at least 1 year between 2008 and 2016 [12]. We excluded critical access hospitals, which do not have a DSH requirement for 340B participation, as well as sole community hospitals and rural referral centers, which face a lower DSH requirement (8%) for 340B eligibility [13]. Because our study focused on general acute care hospitals, we also excluded children’s hospitals and freestanding cancer hospitals, which comprise less than 3% of all 340B hospitals [14]. Investor-owned (i.e. “for-profit”) hospitals are ineligible irrespective of DSH and were excluded from the main analysis.

Small hospitals must report a capped value of 12% DSH even if their actual DSH is higher [15]. In our sample, 40.7% of hospital-year observations (N = 11,361) were subject to the DSH cap. We therefore calculated “true” DSH for all hospitals using formulas published by the Centers for Medicare & Medicaid Services [15]. Among uncapped hospitals in our sample, reported and calculated DSH had correlation of + 0.996.

We grouped hospitals by true DSH into 1 percentage point bins, with no bins containing 11.75%, and graphed the number of hospitals in each bin just above and below the 11.75% eligibility threshold. If hospitals are adjusting their DSH to gain eligibility, we expect this graph to show a sharp increase in the number of hospitals just above 11.75%. We also used McCrary density tests with true DSH as the running variable to assess whether the observed difference in density of hospitals just below and above the 11.75% threshold was significantly larger than what would be expected by chance alone [16]. Thus, our analysis focuses on bunching in the hospital DSH distribution just above the 340B eligibility threshold at a given point in time.

We conducted several alternate specifications to assess the robustness of findings. First, we repeated the McCrary density test using bandwidths (ranges of data near the cutoff) equal to 1, 3, 5 as well as a mean-standard-error optimal bandwidth calculated using a local polynomial estimator [17]. Because 340B eligibility is not affected by DSH values other than 11.75%, a null hypothesis of no manipulation does not generate falsifiable predictions about the DSH density of hospitals far from the threshold. To test for comparable changes in the distribution of DSH at points other than the 11.75% threshold, we repeated the analysis using placebo DSH cutoffs ranging from 6.75% to 16.75%. Third, we conducted a placebo analysis using data from investor-owned hospitals, which are ineligible for 340B irrespective of DSH. Finally, since DSH depends partly on Medicaid inpatient days, an increase in the number of hospitals above the threshold might simply reflect expansions of eligibility for Medicaid to low-income, non-disabled adults implemented in many states starting in 2014 under the Affordable Care Act (ACA). To examine whether this accounted for our findings, we repeated the analysis after dropping states that implemented Medicaid eligibility expansions.

### Results

**Fig. 1:**
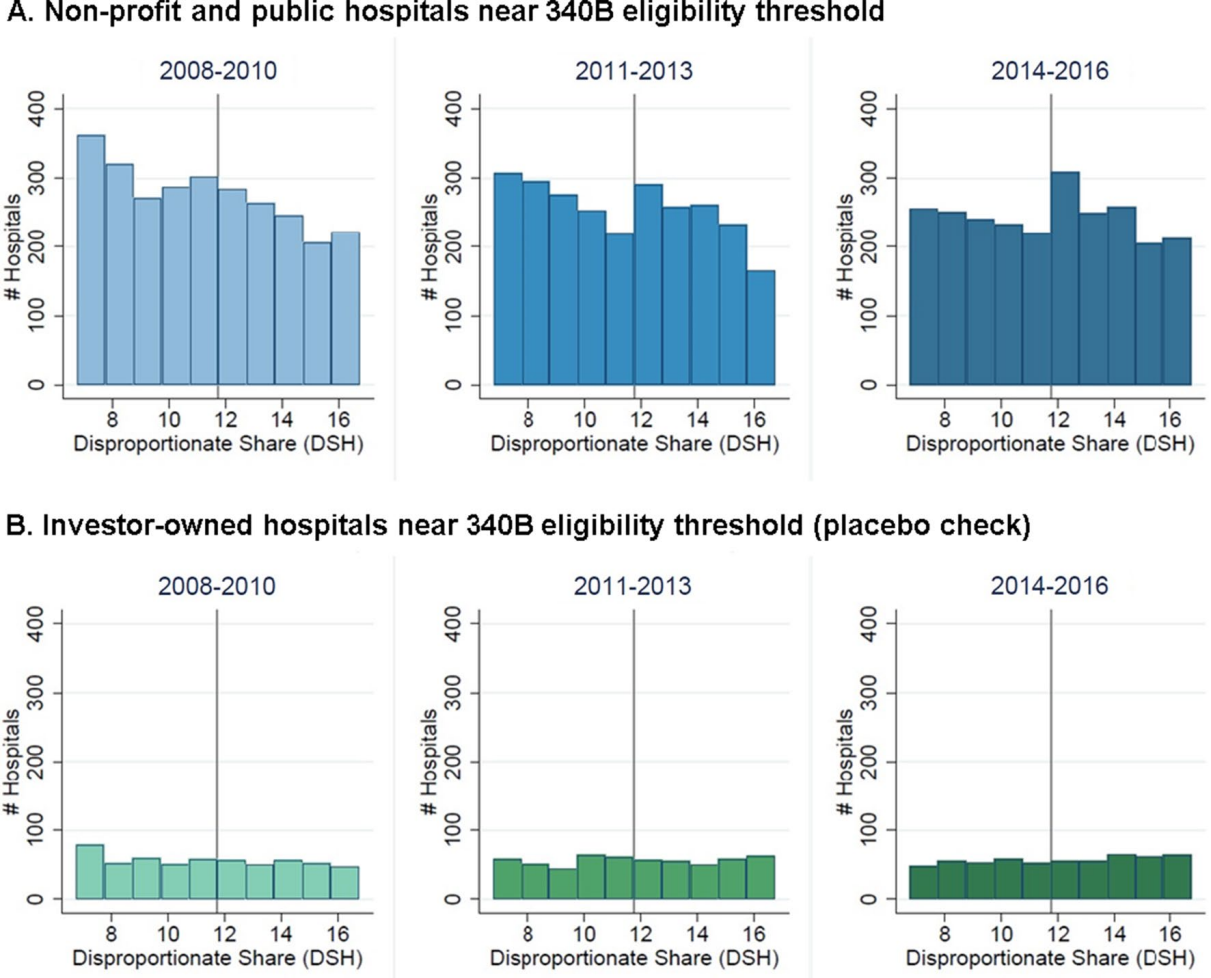
Number of Hospitals near the 340B Eligibility Threshold. Vertical line indicates 340B eligibility threshold (DHS = 11.75%). Hospitals are grouped into 1 percentage-point bins, with no bin containing DSH = 11.75%. Public and non-profit hospitals with DSH greater than 11.75% are eligible for 340B; investor-owned hospitals are not eligible for 340B irrespective of DSH

Figure 1 presents histograms of the DSH variable among public and non-profit hospitals, compared with investor-owned hospitals, which were never eligible for 340B. In 2014–2016, the number of hospitals just above the threshold increases by 41% compared to the number just below it. Prior to 2014, this pattern was much less pronounced.

McCrary density tests found a significant increase just above the 340B eligibility threshold in 2014–2016 (p < 0.01). This finding was present for all bandwidths examined. From 2011–2013, the statistical significance of the change at the threshold was sensitive to the bandwidth used; the change was statistically significant (p < 0.01) for the optimal bandwidth (4.0 below the threshold and 4.3 above) as well as bandwidths of 2 and 5, but not for the bandwidth equal to 1. The McCrary density test did not identify significant changes in the number of hospitals at the 340B eligibility threshold prior to 2011.

**Fig. 2:**
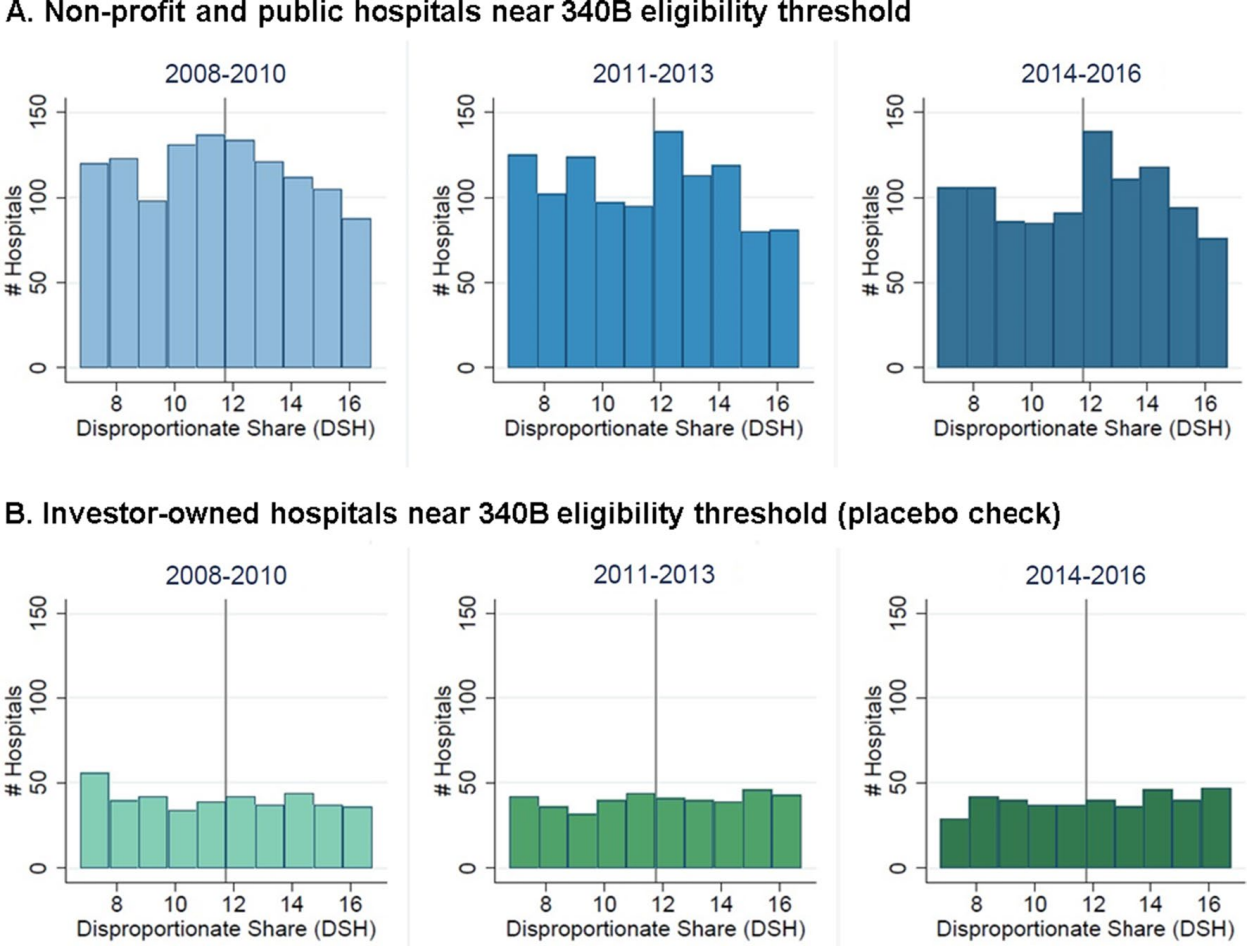
Number of Hospitals Near the 340B Eligibility Threshold in States that did not Expand Medicaid. Vertical line indicates 340B eligibility threshold (DHS = 11.75%). Sample includes hospitals in states that expanded Medicaid following the Affordable Care Act. Hospitals are grouped into 1 percentage-point bins, with no bin containing DSH = 11.75%. Public and non-profit hospitals with DSH greater than 11.75% are eligible for 340B; investor-owned hospitals are not eligible for 340B irrespective of DSH

Sensitivity checks suggested the robustness of findings. We found no comparable evidence of changes in the number of hospitals at cutoffs other than 11.75%. There was no significant change in the number of hospitals at the 11.75% threshold for the investor-owned hospitals, a group of hospitals that is ineligible for the 340B program. Additionally, our findings were robust to dropping states that expanded Medicaid eligibility (Fig. 2). While this suggests Medicaid eligibility expansions do not explain the increase in hospitals just above the threshold in 2014–2016 for the full sample, we cannot rule out the possibility that Medicaid expansions impacted hospitals in non-expansion states that are located near the border of an expansion state.

### Discussion

This paper documents an increase in the number of hospitals whose DSH is just above the 340B eligibility cutoff. This increase at the cutoff is significantly larger than what would be expected by chance alone in 2014–2016; there is suggestive evidence of an increase in 2011–2013, and no evidence of an increase prior to 2011. These patterns are consistent with a hypothesis that some hospitals adjusted their DSH to gain 340B eligibility, although this is a fairly recent phenomenon.

The possibility of DSH adjustment was examined in a previous study that used a regression discontinuity design (RDD) to estimate the effect of the 340B program on hospital-physician consolidation and the administration of non-oral drugs by hospital-owned facilities [18]. In particular, the RDD estimates would be invalid if hospitals strategically adjust the factors (i.e. DSH) that determine 340B program eligibility [19]. Desai and McWilliams (2018) used data from 2008–2012 [18], and our results for 2008–2010 are consistent with theirs. However, for 2011–2013 (i.e., 2 year overlap with the previous study), we found that the statistical significance of McCrary density tests was sensitive to bandwidth. Desai and McWilliams (2018) did not find evidence of manipulation, however they used graphical analysis and analyzed changes in the same hospital over time rather than McCrary density tests [18]. From a methodological standpoint, our results suggest that RDD may not be a valid approach for studying the 340B program, particularly after 2014 when we find the strongest statistical evidence consistent with strategic behavior.

Although we cannot determine the mechanism that drives our results, several possibilities exist. First, hospitals may have increased their DSH through better record keeping without substantive changes in patient mix. Better record keeping would be consistent with observed high adoption rates of electronic health records by hospitals following the passage of the ACA [20]. Alternatively, administrators at hospitals near the eligibility threshold may have accepted more Medicaid or dual eligible patients in order to move their DSH above the 11.75% cutoff. Finally, it is possible that some hospitals near the eligibility cutoff misreported their DSH or the underlying patient mix to gain access to the 340B program.

Our data are relevant to ongoing policy discussions. While there is some oversight of the 340B program, it is limited, with only 200 covered entities receiving audits annually [21]. The GAO has issued calls for improved reporting and oversight in the 340B program [5, 21]. Our data support these calls for additional oversight and suggests attention could be focused on hospitals near the eligibility threshold.

## Limitations

The primary limitation of our study is related to interpretability of results. Our findings of more hospitals above the threshold than would be expected by chance only applies to hospitals “near” the eligibility threshold. For example, in 2016 there were 2189 hospitals in our main sample, with 185 having true DSH 2 percentage points or less above the threshold. Thus, based on a back-of-the-envelope calculation, at most roughly 8% of hospitals may have adjusted their DSH to shift just above the 340B eligibility threshold. While strategic behavior to adjust DSH may be more widespread (i.e., hospitals may adjust DSH well beyond the threshold), available statistical tests do not allow us to check for this possibility. Finally, while 340B has been available since 1992, the costs and benefits tied to 340B participation by hospitals may have shifted over time. While this is an important issue, it is outside the scope of this paper and an area for future research.

## Data Availability

Please contact the corresponding author (Karen Mulligan) to request a copy of the data used for this research.

## Abbreviations

ACA: Affordable Care Act
DSH: Disproportionate share
GAO: Government Accountability Office
HCRIS: Healthcare Cost Report Information System
